# Lipid composition in a strain of *Bacillus subtilis*, a producer of iturin A lipopeptides that are active against uropathogenic bacteria

**DOI:** 10.1007/s11274-016-2126-0

**Published:** 2016-08-23

**Authors:** Przemysław Bernat, Katarzyna Paraszkiewicz, Paulina Siewiera, Magdalena Moryl, Grażyna Płaza, Joanna Chojniak

**Affiliations:** 1 Department of Industrial Microbiology and Biotechnology, Faculty of Biology and Environmental Protection, University of Lodz, Banacha Street 12/16, 90-237 Lodz, Poland; 2 Department of Immunobiology of Bacteria, Faculty of Biology and Environmental Protection, University of Lodz, Banacha Street 12/16, 90-237 Lodz, Poland; 3 Department of Environmental Microbiology, Institute for Ecology of Industrial Areas, Kossutha Street 6, 40-844 Katowice, Poland

**Keywords:** *Bacillus subtilis*, Iturin, Lipidomics, Lipopeptides, Phospholipids, Uropathogens

## Abstract

**Abstract:**

Urinary tract infections are a common disease in humans. Therefore, new methods are needed to destroy biofilms that are formed by uropathogens. Iturin A lipopeptides (LPs) C14 and C15 are potent biosurfactants synthetized by the *Bacillus subtilis* I′1a strain. The biological activity of extracted LPs was confirmed by examining extracts from I′1a cultures against uropathogenic bacteria that had been isolated from biofilms on urinary catheters. Compared with cultures of DSM 3257, which produce surfactin at a relatively low level, the extract obtained from strain I′1a exhibited a greater inhibitory effect against both planktonic and sessile forms of *Escherichia coli*, *Serratia marcescens*, *Enterobacter cloacae*, *Proteus mirabilis*, *Citrobacter freundii* and *Enterococcus faecalis.* Moreover, cyclic LP biosurfactants may disturb the integrity of cytoplasmic membranes; therefore, we investigated the effects of synthetized LPs on fatty acids and phospholipids of *B. subtilis*. LPs and lipids were analyzed using GC–MS, LC–MS/MS and MALDI-TOF/TOF techniques. Compared with *B. subtilis* DSM 3257, membranes of the I′1a strain were characterized by an increased amount of anteiso fatty acids and a ten-fold higher ratio of phosphatidylglycerol (PG)-to-phosphatidylethanolamine (PE). Interestingly, in cultures of *B. subtilis* DSM 3257 supplemented with LP extracts of the I′1a strain, the PG-to-PE ratio was fourfold higher, and the amount of anteiso fatty acids was also increased.

**Graphical Abstract:**

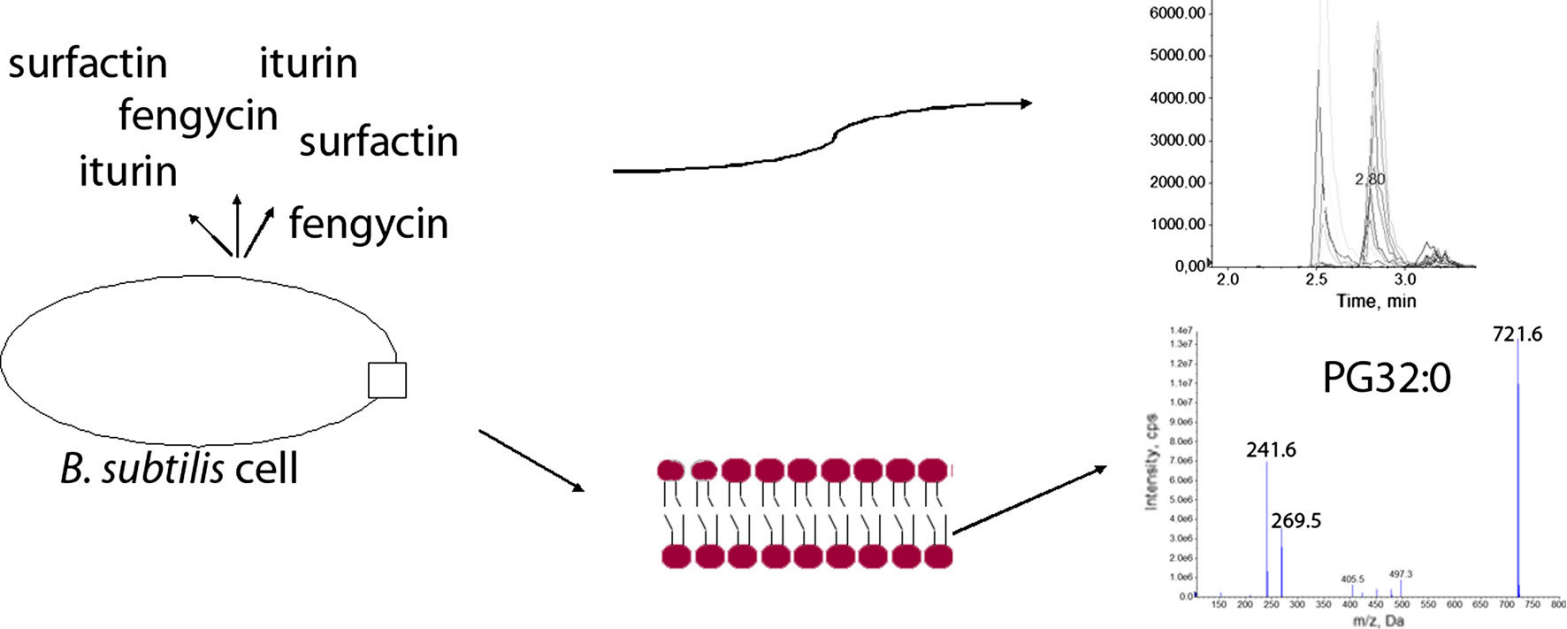

## Introduction

*Bacillus* strains are known to be producers of cyclic lipopeptide (LP) biosurfactants which are mainly represented by members of the surfactin, iturin and fengycin families. These LPs have recently been reviewed in detailed, including their classification, structural diversity, functional and biological properties, roles in the growth of the producing micro-organisms, interactions with coexisting organisms and application potential (Ongena and Jacques 2008; Hamley 2015; Meena and Kanwar 2015; Mnif and Ghribi 2015; Wang et al. 2015). Biomedical applications of bacilli LPs are based on the ability of these compounds to interfere with flagella development, affect bacterial adhesion, inhibit biofilm formation and disrupt pre-formed biofilms (Rivardo et al. 2009; do Valle Gomes and Nitschke 2012; Janek et al. 2012).

Many bioactive properties of surfactin, iturin and fengycin result from the capability of these molecules to disturb the structures and functions of biological membranes, which leads to increased membrane permeability. The mode of surfactin interactions may be strongly concentration-dependent. According to Deleu et al. (2013), below a critical micelle concentration (CMC, 3 µM), surfactin inserts into the boundary between gel and fluid lipid domains without inducing global morphological changes. By contrast, at concentrations close to the CMC, it solubilises the fluid phospholipid phase, and at higher concentrations both the fluid and rigid bilayer structures are dissolved into mixed micelles. Iturin A is a less potent biosurfactant than surfactin, and its CMC is ~25 μM (Aranda et al. 2005).

Previously, we described the bacterial strain *B. subtilis* I′1a as a surfactin, iturin and fengycin co-producer (Plaza et al. 2015). It was also found that lipopeptides extracted from cultures of these bacteria had a strong antimicrobial effect on uropathogenic bacteria, including effects on planktonic growth, and the processes of biofilm formation and dislodging (Moryl et al. 2015). In this present study, we carried out a more detailed elucidation of the structure of LPs produced by the I′1a *B. subtilis* strain. Moreover, we sought to determine whether the lipopeptides could alter bacterial fatty acid and phospholipid composition. The strain *B. subtilis* DSM 3257 (which only synthesized surfactin at a low level) was used to compare the lipid composition and anti uropathogenic activities.

## Materials and methods

### Reagents

Phospholipid standards: 1,2-Dimyristoyl-sn-glycero-3-phospho-rac-(1-glycerol) (sodium salt), 1,2-dilauroyl-sn-glycero-3-phosphoethanolamine, 1,2-dimyristoyl-sn-glycero-3-phosphate (sodium salt), 1,2-dimyristoyl-glycero-3-phosphocholineand cardiolipin solution from a bovine heart were purchased from Avanti^®^ Polar Lipids, Inc. (Alabaster, AL, USA) or Sigma-Aldrich.3-(4,5-dimethylthiazol-2-yl)-2,5-diphenyltetrazolium bromide (MTT) and 2,5-dihydroxybenzoic acid (DHB) were purchased from Sigma-Aldrich, tryptone soya broth (TSB) was from BTL (Lodz, Poland). Surfactin and iturin A were obtained from Sigma-Aldrich. The other chemicals came from J.T. Baker, Fluka and POCh (Gliwice, Poland). All the chemicals were high purity grade reagents.

### Characterization and culture of *B. subtilis* strains

Throughout the study, two strains of *Bacillus subtilis* were used. The first strain, *B. subtilis* DSM 3257, had a proven ability to synthesize surfactin and was obtained from the Leibnitz Institute DSMZ—German Collection of Micro-organisms and Cell Cultures. The second strain, *B. subtilis* I′1a, was generously supplied by the Institute for Ecology of Industrial Areas (Katowice, Poland) and was an isolate obtained from the sludge of a 100-year-old oil refinery in Czechowice-Dziedzice (Poland). Taxonomic identification and preliminary studies of biosurfactant production by strain I′1a have been described previously (Berry et al. 2006; Plaza et al. 2006; Plaza et al. 2010; Plaza et al. 2011).

Strains were stored at (−70 °C), as stocks of 24-h-old cultures in Luria–Bertani (LB) medium (Fluka, Germany), pH 7.0, containing in g/L casein peptone (10.0 g); yeast extract (5.0 g) and NaCl (5.0 g) were supplemented with 20 % (v/v) glycerol, before use in this study.

*B. subtilis* cultures were grown in LB medium for 24 h on an orbital shaker (140 rpm) at 28 °C. The resulting seed cultures were diluted with LB medium to OD = 0.8 (at λ = 600 nm) and used in a 3 mL volume to inoculate 97 mL LB medium. Cultures were incubated in 300 mL Erlenmeyer flasks for 72 h under the conditions described above. Samples of 24-, 48- and 72-h-old cultures were used for measurements of optical density and then were centrifuged (10,000×*g*, 10 min). The resulting supernatants were used for surface tension and lipopeptides assessments. Fatty acids and phospholipids were isolated from the remaining biomass.

### Surface tension (ST) measurements

To study the surface activity of biosurfactants produced by the *B. subtilis* strains, supernatant samples of the centrifuged cultures were measured for ST using a Du Nöuy ring with a tensiometer SIGMA 702 (Attension). ST measurements were carried out at room temperature after dipping a platinum ring in the solution for enough time to attain equilibrium conditions. To calibrate the instrument, the ST of pure water was measured. Measurements were repeated at least three times, and an average value was used to express the surface activity of each sample. Attension software was used to analyse all data.

### Isolation and quantitative analysis of LPs by liquid chromatography–mass spectrometry (LC–MS/MS)

LPs isolation was performed as described by Plaza et al. (2015) with several modifications. Supernatants from a culture sample with a volume of 10 mL were acidified with 6 N HCl to pH 2 and stored overnight at 4 °C. The precipitate that formed was collected by centrifugation (10,000×*g*, for 20 min, at 4 °C) and later mixed with 10 mL distilled water; the pH of the sample was adjusted to 7.0 using 1 N NaOH. Next, a 10 mL ethyl acetate and methanol mixture (4:1, v/v) was added and each sample was vigorously shaken for 30 min. LPs extraction was repeated three times. Anhydrous sodium sulfate was added to the collected organic phase, and after filtration, solvent was evaporated. Extracts examined by LC–MS/MS were dissolved in methanol (2 mL). Surfactin analysis was performed using an Agilent 1200 LC (Santa Clara CA, USA) system with a 3200 QTRAP mass spectrometer (AB Sciex, Framingham, MA, USA) equipped with an ESI source. Samples (5 μL) were injected onto an Allure^®^ PFP Propyl column (50 mm × 2.1 mm, 5 μm particle size; Restek, Bellefonte, PA, USA) and maintained at 40 °C. The mobile phase consisted of water (A) and methanol (B), which were both supplemented with 2 mM ammonium formate and 0.2 % formic acid. The run time was 8 min with the solvent gradient was initiated at 60 % B. After 1 min, the amount of B was increased to 100 % during the next minute and was maintained at 100 % for four additional minutes before returning to the initial solvent composition over the next 2 min. The flow rate was 600 mL/min.

MS/MS data were collected and processed using Analyst™ v1.5.2 software (AB Sciex, Framingham, MA, USA). Quantitative lipopeptides analyses were performed for surfactin and iturin A standards (Sigma–Aldrich) and QTRAP 3200 in multiple reaction monitoring positive ionization mode (MRM). The electrospray source was operated at a temperature of 600 °C and voltage of 5500 V. The monitored MRM pairs were *m/z* 1030–391, 1044–391, 1058–391 and 1072–391 for sodiated molecules [M + Na]^+^ of the surfactin homologues C13, C14, C15 and C16, respectively. For sodiated ions of homologues C14, C15 and C16 of iturin A, the MRM pairs were *m/z* 1065.6/293, 1079.6/237 and 1093.6/1093, respectively.

### Lipopeptide identification

A MALDI-TOF/TOF–MS spectrometer AB SCIEX 5800 TOF/TOF System (AB Sciex) was used for more detailed elucidation of the structures of LPs produced by the *B. subtilis* strains that were studied. A mixture consisting of 0.5 μL obtained LPs extract (diluted in 2 mL methanol) and 0.5 μL matrix solution (containing 10 mg/mL DHB dissolved in acetonitrile) was deposited onto the MALDI target. MALDI-TOF/TOF analyses were conducted in positive ionization and reflector mode by accumulating 1000 laser shots in the range *m/z* 900–2000 to one mass spectrum. Uniform, continuous, and random stage motion at 800 µm/s was selected for data acquisition at a fixed laser intensity of 3500 (instrument-specific units) and a 400 Hz pulse rate. The ten most intense signals per spot were selected for automated MS/MS measurements. MALDI-TOF/TOF spectra were acquired by accumulating ten spectra (200 shots each) at a 1000 Hz pulse rate with a mass range adjusted to a *m/z* value of a respective precursor. A continuous stage-motion of 800 µm/s was selected at a fixed laser intensity of 5000 (instrument-specific units).

### Lipid extraction

Lipids were extracted according to a modified Bligh and Dyer method (1959). Cells from 24-, 48- and 72-h-old cultures were harvested by centrifugation (5000×*g*, 10 min, 4 °C) (MPM, Poland). Subsequently, 1 mL 0.89 % NaCl and 3.75 mL CHCl_3_–MeOH mixture (1:2, v/v) were added and the biomass was crushed with a ball mill PM 200 (Retsch, Germany). The homogenate was collected and then 1.25 mL chloroform and 1.25 mL H_2_O were added. Next, vials were vortexed for 2 min and centrifuged. The lower organic phase was collected, treated with anhydrous sodium sulphate, and evaporated under reduced pressure. Residues were re-dissolved in 2 mL methanol/chloroform solution (2:1, v/v) and stored at −20 °C for subsequent analysis.

### Determination of phospholipid molecular species by LC–MS/MS

Phospholipid measurements were performed using an Agilent 1200 LC system and a 4500 QTRAP mass spectrometer (AB Sciex) with an ESI source. For reversed-phase chromatographic analysis, 5 µL lipid extract was injected onto an Allure^®^ PFP Propyl column (50 mm × 2.1 mm, 5 µm particle size; Restek). The gradient profile of the mobile phase that consisted of water and methanol is presented in supplementary Table S1. The column temperature was maintained at 40 °C with a flow rate of 600 mL/min. Prior to use of the column, a blank gradient was run. Nitrogen was used as a nebulizer, heater, and curtain gas with the pressure set at 50, 60, and 25 psi, respectively. The electrospray ionization voltage was set to −4500 V, and the temperature of the ion source was 600 °C. Data analysis was performed using Analyst^TM^ v1.6.2 software (AB Sciex).

To survey the phospholipid species, information-dependent acquisition (IDA) method, PI → EPI, was used. Spectra were obtained over a range from *m/z* 100–950 and *m/z* 100–1600 for cardiolipin. The EPI scan rate was 10,000 amu/s. A scan of the precursor for *m/z* 153, *m/z* 196 or the neutral loss of *m/z* 87 was used to detect the phospholipid subspecies. The mass spectra of phosphatidic acid (PA), phosphatidylglycerol (PG), lysyl-phosphatidylglycerol (LPG), phosphatidylethanolamine (PE) and cardiolipin (CL) species showed ions that corresponded to the deprotonated molecules [M–H]^−^.

Negative ion matrix-assisted laser ionization/desorption time-of-flight tandem mass spectrometry (MALDI-TOF/TOF MS, AB Sciex 5800) experiments were also performed for the identification of phospholipids. Briefly, lipids prepared as above were spotted (0.7 µL) directly onto a MALDI sample plate, followed by 0.7 µL 30 mg/mL MALDI matrix dissolved in methanol. MALDI analyses were conducted in negative ionization and reflector mode by accumulating 1000 laser shots at a range of *m/z* 500–1600 to one mass spectrum. The ten most intense signals per spot were selected for automated MS/MS measurements.

Based on the product ion and precursor ion analyses of the head groups and fatty acyl chains, a comprehensive list of MRM transitions was generated. The signal intensity of each MRM value was normalized to the sum of MRM intensities of all species.

### Fatty acid analysis

Fatty acid methyl esters (FAMEs) were prepared according to a method previously described by Bernat and Długoński (2007) with some modifications. Bacterial cells harvested as above from 48-h-cultures were placed into Pyrex glass tubes and 4 mL solution that consisted of methanol:toluene:H_2_SO_4_ (30:15:1, by volume) was added to each tube. After 18 h incubation at 50 °C, samples were cooled to room temperature and FAMEs were extracted twice with 4 mL hexane. Extracts were dried over anhydrous sodium sulphate and evaporated. Then, 1 mL hexane was added and the samples were analyzed using gas chromatography (GC).

FAMEs analysis was performed using an Agilent Model 7890A gas chromatograph that was equipped with a 5975C Mass Detector. Separations were carried out in a capillary column HP 5 MS (60 m × 0.25 mm id × 0.25 mm ft). The column temperature was maintained at 60 °C for 3 min, then was increased to 215 °C at 6 °C/min, followed by an increase to 250 °C at 2 °C/min and finally to 280 °C at 20 °C/min. The column temperature was then maintained at 280 °C for 10 min. Helium was used as a carrier gas at a flow rate of 1 mL/min. The injection port temperature was 275 °C. A 1.6 µL volume of solution was injected into a split injector. Bacterial fatty acids were identified by comparison with the retention times of the authentic standards (Sigma, Supelco) or based on the mass spectra and were expressed as a percentage of total fatty acids.

### Tolerance of the *B. subtilis* DSM 3257 strain to I′1a lipopeptides extract

LP extract from I′1a culture obtained as above was diluted in methanol (the methanol concentration in the samples did not exceed 2 % and had no effect on the growth of the bacteria that we tested) and was added to *B. subtilis* DSM 3257 submerged cultures (prepared as described above). Samples of 24-, 48- and 72-h-old cultures were used for measurements of the optical density and phospholipid profiles.

### Antimicrobial activity of LP extracts

The antimicrobial activities of LPs extracts obtained from *B. subtilis* DSM 3257 and I′1a cultures were tested against 10 uropathogenic strains belonging to the following 6 species: *Escherichia coli, Serratia marcescens, Enterobacter cloacae, Proteus mirabilis, Citrobacter freundii* and *Enterococcus faecalis*; these species were isolated from biofilms formed on urinary catheters in long-term catheterised patients. The ability of the uropathogenic strains that were tested to form biofilm on abiotic surfaces, as well as their resistance to antibiotics, had previously been characterized (Moryl et al. 2013). Bacterial strains were stored at (−70 °C) as stocks of 24-h-old cultures using Luria–Bertani (LB) medium (Fluka, Germany) pH 7.0, supplemented with 10 % (v/v) dimethyl sulfoxide (DMSO).

Before the application in antimicrobial assays, LP extracts were diluted in methanol and then mixed with phosphate-buffered saline (PBS), pH 7.2, to obtain a LPs concentration of 40 mg/L in samples. The methanol concentration in the samples did not exceed 1 % and had no effect on the growth of the bacteria that were tested.

The LP-inhibitory effects on the growth of uropathogen planktonic forms were tested using two different methods: a modified microdilution assay (Rajaram et al. 2010) and an agar diffusion test (Diep et al. 2000). Examinations of LP extracts antimicrobial activity in biofilms were performed using the modified microdilution method in flat-bottomed microplates (Janek et al. 2012). Before applying them in antimicrobial tests, uropathogens were cultivated in tryptone soya broth (TSB) at 37 °C, for 18 h and then the culture samples were diluted in TSB to a cell concentration of 10^7^ CFU/mL. For the agar diffusion test, 1 mL prepared bacterial suspension was deposited on Mueller–Hinton agar. Next, LP extracts with a target concentration of 20 mg/L were transferred to the plates. Plates were incubated at 37 °C for 24 h. Then, the diameter of microbial growth inhibition (i.e., the halo zone) was measured.

The microdilution method and studies biofilm formation were carried out as described by Moryl et al. (2015).

### Statistical analysis

Experimental data represent means of at least three independent experiments. Student’s *t* test was used to determine the statistical significance of differences between means.

## Results

### Comparison of *B. subtilis* growth kinetics and lipopeptide production

**Fig. 1:**
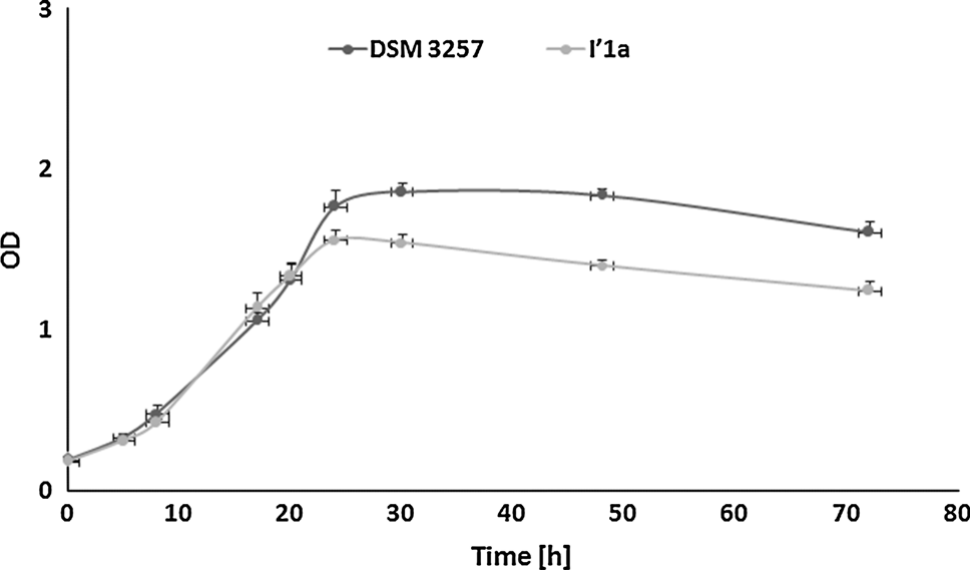
Growth of *B. subtilis* species during 72-h cultivation in LB medium. *Error bars* represent standard deviation (*n* = 3)

As shown in Fig. 1, the cultures were grew almost without any visible lag phase, probably because of the same conditions were used for inoculum and second-step culture maintenance. During the exponential phase, specific growth rates were 0.125 and 0.11 h^−1^ for cultures of DSM 3257 and I′1a, respectively. The growth kinetics of the cultures that were studied were very similar up to 20–24 h, when the stationary phase was reached. In cultures of the I′1a strain, the cell density reached a maximal value of 1.49 at 24 h and then gradually decreased to 1.1 (as measured at 72 h). By contrast, in the culture of DSM 3257, the maximal cell density was slightly higher (1.86) and was obtained later (at 30 h), after which it declined to 1.6 by the end of the cultivation.

The surface activity was analysed via ST measurement using cell-free-broth samples obtained from 24-, 48- and 72-h-old *B. subtilis* cultures. The strongest reduction in the ST was observed in samples cultured for 48 h. For the DSM 3257 and I′1a strains, the values were 53.7 and 31.7 m/Nm, respectively.

### Antibacterial activity of *B. subtilis* extracts

The agar diffusion method was used to study the effect of LP extracts on planktonic forms of uropathogens on solid medium, which demonstrated the strong bacteriostatic effects of extracts obtained from the *B. subtilis* I′1a culture (Table 1, column A). All tested bacteria were found to be sensitive to this extract, with a halo zone size that ranged from 5 to 19 mm. For the *B. sublilis* DSM 3257 extract, there were no changes (zones) on the Mueller–Hinton plates. The sensitivity of uropathogen planktonic forms to tested *B. subtilis* LP extracts obtained by a dilution assay is shown in Table 1, column B. Increased activity against the bacteria was exhibited by those compounds produced by *B. subtilis* I′1a. Extracts from cultures of this strain inhibited the growth of 9/10 tested uropathogens, and the average reduction in the absorbance was ~92 %. *B. subtilis* DSM 3257 extracted products affected the growth of only one uropathogenic strain, *E. coli* 56, with a 60 % reduction in absorbance.

**Table 1:** Influence of LPs extracts obtained from 48-h cultures of *B. subtilis* DSM 3257 and I′1a on uropathogen growth, based on: (A) diameter of the inhibition zone (mm) in an agar diffusion test, (B) inhibition of planktonic cell growth (%), (C) inhibition of biofilm formation (%) and (D) mature biofilm reduction (%)

Uropathogenic strains	Antimicrobial activity							
	DSM 3257 LPs extract				I′1a LPs extract			
	A	B	C	D	A	B	C	D
*E. coli* 9	0	0	56.53 ± 14.55	46.07 ± 6.13	5 ± 2	92.96 ± 0.75	87.93 ± 2.29	50.89 ± 3.30
*E. coli* 56	0	59.58 ± 3.21	0	0	17 ± 5	80.42 ± 9.89	66.84 ± 4.83	38.17 ± 2.11
*E. coli* 84	0	0	0	0	12 ± 3	86.31 ± 0.99	74.29 ± 4.49	75.92 ± 2.85
*S. marcescens* 19	0	0	66.65 ± 2.65	0	16 ± 5	96.80 ± 0.23	98.47 ± 0.43	79.09 ± 2.73
*S. marcescens* 23	0	0	64.82 ± 5.59	6.83 ± 2.65	15 ± 3	91.91 ± 0.48	87.82 ± 4.24	24.67 ± 2.35
*E. cloacae* 30	0	0	45.40 ± 5.17	0	20 ± 6	93.18 ± 0.83	75.30 ± 12.31	40.54 ± 4.15
*E. cloacae* 64	0	0	18.81 ± 12.46	0	18 ± 5	42.64 ± 6.98	10.05 ± 9.0	23.79 ± 4.38
*P. mirabilis* 70	0	0	0	0	19 ± 4	98.73 ± 0.03	71.27 ± 5.0	9.44 ± 1.88
*C. freundii* 16	0	0	0	3.24 ± 2.39	5 ± 1	93.48 ± 0.59	46.54 ± 6.28	0
*E. faecalis* 9	0	0	0	0	10 ± 3	96.07 ± 0.48	0	0

Data represent mean ± SD

The tested *B. subtilis* extract exhibited an ability to inhibit biofilm formation by uropathogenic strains (Table 1, column C). Higher activity against the studied micro-organisms was exhibited by LP extracts from *B. subtilis* I′1a as a consequence of inhibition of the growth of seven uropathogenic strains by an average of 80.27 %. The LP extract of *B. subtilis* DSM 3257 showed a lower ability to affect biofilm formation and caused an average of 65.73 % reduction in biofilm biomass among the various bacterial strains that we tested.

Our studies of the influence of the LP extracts on mature biofilms (by the evaluation of the degree of biofilm dispersion) are presented in Table 1, column D. We found that the biofilms produced by *E. coli* 9, *E. coli* 84 and *S. marcescens* 19 were sensitive to the compounds that were present in the *B. subtilis* I′1a extracts with the biofilm reduction of ~68.63 %. There was no significant effect of *B. subtilis* DSM 3257 extracts on the process of biofilm destruction. Thus, we concluded that the examined extracts had a lower impact on biofilm dislodging than on biofilm formation.

### Mass spectrometry analysis of *B. subtilis* lipopeptides

**Fig. 2:**
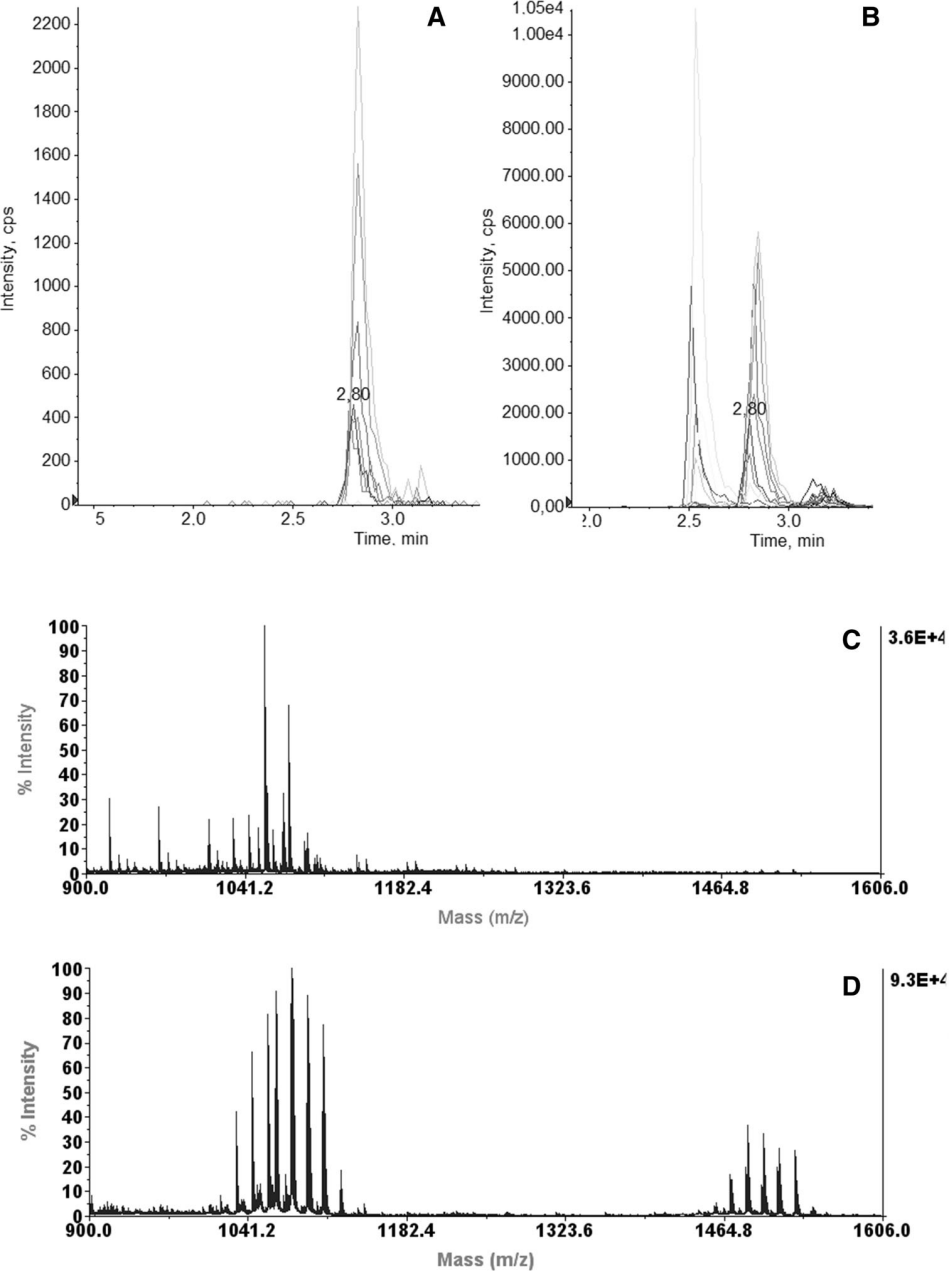
MRM chromatograms and MALDI-TOF spectra of lipopeptides isolated from *B. subtilis* DSM 3257 (**a**, **c**) and I′1a (**b**, **d**). All extracts were obtained from a similar culture volume. The RT for surfactin, iturun A and fengycin homologues was 2.8, 2.53 and 3.2, respectively

LC–MS/MS chromatograms of the analyzed *B. subtilis* LP extracts revealed differences in LP production between the strains that we examined (Fig. 2). In all samples, four peaks of surfactin homologues were revealed at a retention time of 2.79, which corresponded to sodiated molecules [M + Na]^+^*m/z* 1030, 1044, 1058 and 1072 in positive modality. Approximately 2.5 min, peaks of iturins [M + Na]^+^*m/z* 1065, 1079 and 1093 were found, while in the range of 3.0–3.5 min, peaks of fengycin lipopeptides were observed. Our findings indicated that for strain I′1a, the predominant ion mass peak in positive ion mode was a homologue of C15 iturin A, while those of surfactin homologues were less visible.

The surfactin content increased between 24 and 72 h in the DSM 3257 and I′1a cultures from 2.5 to 4.0 and from 5.1 to 9.2 mg/L^−1^, respectively (Table 2). In all analysed samples, surfactin was presented as a mixture of four homologues (from C13 to C16), among which the C14 and C15 variants were predominant and together comprised up to 90 % of the total analysed surfactin content (data not shown).

**Table 2:** Surfactin and iturin A concentrations in cultures of *B. subtilis* DSM 3257 and I′1a grown in LB medium

*B. subtilis* strain	Surfactin concentration (mg/L)		
	24 h	48 h	72 h
DSM 3257			
Surfactin	2.46 ± 0.12	3.64 ± 0.3	4.04 ± 0.22
Iturin A	n.d	n.d	n.d
I′1a			
Surfactin	5.24 ± 0.48	7.92 ± 0.3	8.54 ± 0.64
Iturin A	19.74 ± 0.85	48.31 ± 2.32	55.96 ± 1.17

Data represent mean ± SD

*n.d.* not detected

By contrast, a high amount of iturin A was noted in the I′1a strain. The concentration of lipopeptide increased during bacterial incubation, and reached 77 mg/L at 72 h.

To precisely identify metabolites structures, especially for the amino acid sequences of the peptide portion of molecules, MALDI TOF/TOF was applied. Intense signals in the *m/z* ranges of 900–1150 and 1400–1600 were obtained in the MALDI-TOF–MS spectra of lipopeptide extracts. In the I′1a extracts, an abundance of iturins containing C14 and C15 fatty acid chains, with a series of H^+^, Na^+^, and K^+^ adduct ions at *m/z* 1043, 1065, and 1081 and at *m/z* 1057, 1079, and 1095, respectively, were observed. In extracts of that strain, sodiated molecules of fengycin ions containing C15, C16 and C17 fatty acid chains at *m/z* 1457, 1471, 1485, 1513 and 1527 were also identified (Fig. 2).

**Fig. 3:**
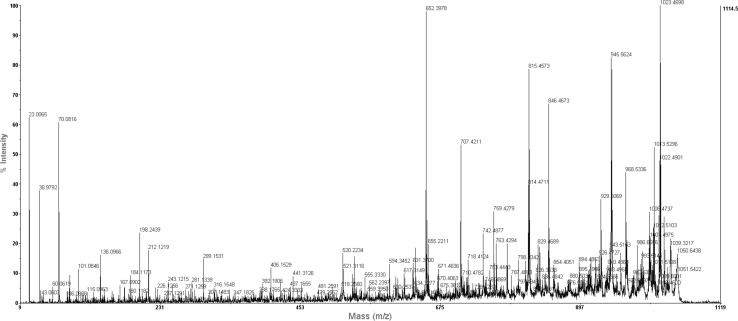
MALDI-MS/MS spectrum of [M + H]^+^ ion at *m/z* 1057.5 from *B. subtilis* I′1a

From the spectrum of the ion *m/z* 1057 identified in the I′1a extract, an ion at *m/z* 198 was detected and found to be the immonium ion of the β-amino acid (H_2_N^+^=CHC_12_H_25_). The lower-mass region of the MS/MS spectra, the indicated peaks corresponded to immonium ions (H_2_N^+^=CH–R) of the individual constituent amino acids—Ser (*m/z* 60), Pro (*m/z* 70), Gln (*m/z* 84), Asn (*m/z* 87) and Tyr (*m/z* 136). The main linear acylium ions of iturin may have been Pro-Asn-Ser-βAA-Asn-Tyr-Asn-Gln-CO^+^. Together, these findings confirmed that the compound with a molecular weight of *m/z* 1057 was a homologue of C15 iturin A (Fig. 3).

### Analysis of phospholipids by LC–MS/MS

**Fig. 4:**
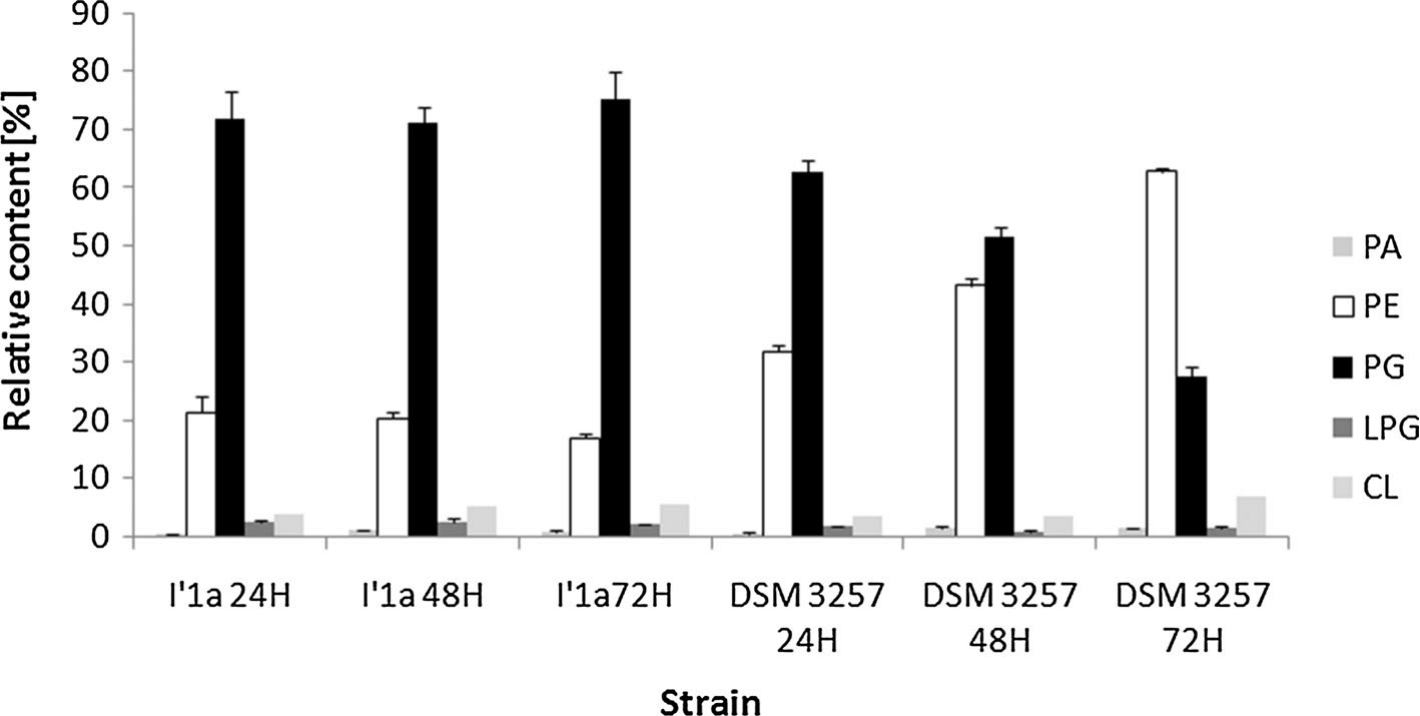
Relative percentage of phospholipid classes measured in *B. subtilis* during 72 h of culture. *PA* phosphatidic acid, *PE* phosphatidylethanolamine, *PG* phosphatidylglycerol, *LPG* lysyl-phosphatidylglycerol, *Cl* cardiolipin. *Error bars* indicate standard deviation (n = 3)

**Fig. 5:**
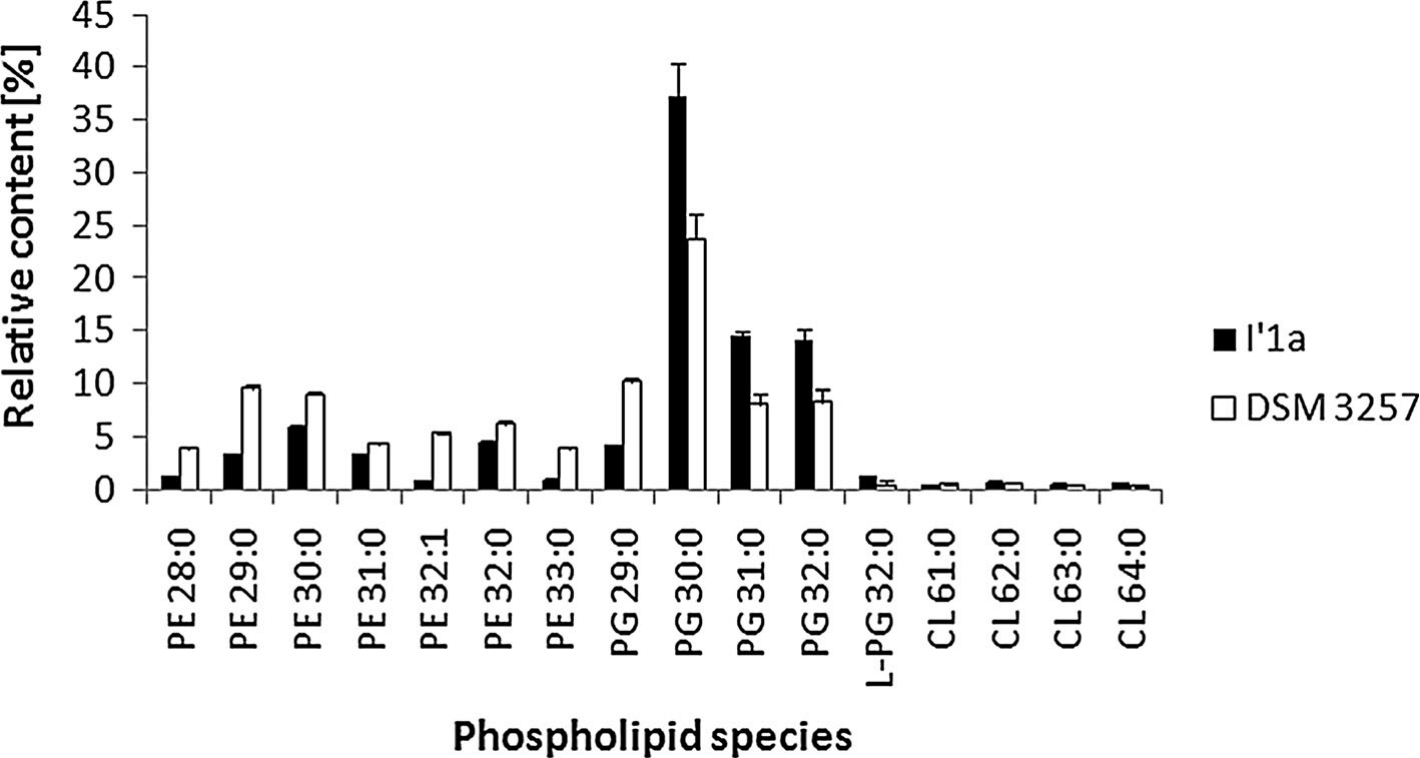
Comparison of phospholipids extracted from 48-h-old *B. subtilis* strains. *PA* phosphatidic acid, *PE* phosphatidylethanolamine, *PG* phosphatidylglycerol, *LPG* lysyl-phosphatidylglycerol, *Cl* cardiolipin. Data indicate mean ± SD (n = 3)

Analyses and quantification of *B. subtilis* phospholipids was carried out after the separation of total lipid extracts by liquid chromatography. To identify the lipids that were present in the lipid extract, MALDI analyses were also performed. All experiments were performed in negative ionization mode to study the fatty acyl chain composition. Based on previously published data (Rezanka et al. 2012), the LIPID MAPS Structure Database and our previous results, we could identify individual molecular species. Examples of some phospholipid mass spectra are shown in Fig. S1 and S2. Table S2 shows assignments of the major *m/z* signals present in lipid extracts of *Bacillus* cells. LC–MS/MS analysis with the formation of [M–H]^−^ ions (Figs. 4, 5) allowed for the identification of PGs, PEs, CLs, LPGs and PAs. Most of the PGs and PEs were saturated fatty acyl chains–C14 and C15. All *Bacillus* strains that we studied had PGs as the main membrane components after 24 h of culture, which made up two-thirds of the total phospholipid fraction. PE was the second most abundant class, which accounted for 21–31 %, followed by CL (3–6 %) and LPG (1.1–2.5 %). The determined phospholipid classes were comparable to previous reports, in which the membrane fraction of PG in *B. subtilis* was reported to be 32 % (Seydlová and Svobodová 2008). Notably, the two studied strains revealed a different phospholipid composition during growth. A comparison samples from DSM 3257 with I′1a cultures, the strain exposed to a high concentration of lipopeptides had a significantly lower level of PE (*P* < 0.01). After 3 days of culture, 16.88 % PE in DSM 3257 and 62.74 % PE in I′1a was observed. Strain DSM 3257 changed its phospholipids profile for the PG-to-PE ratio from 1.98 to 0.44, while in the strain I′1a the ratio changed from 3.38 to 4.45. There were no significant differences observed in the levels of the minor species—PA, CL and LPG (*P* < 0.05).

The relative amounts of particular phospholipid species varied between the two strains (Fig. 5). Each phospholipid profile contained ~30 individual species, which were characterized by the number of carbon atoms within both fatty acid moieties and the degree of saturation. Additionally, MS/MS experiments provided information on individual fatty acid moieties (Fig. 5). Hence, PG(32:0) was composed of not only 16:0/16:0 but also 15:0/17:0. Notably, PGs at *m/z* 693.5 (30:0), *m/z* 707.5 (31:0) and *m/z* 721.5 (32:0) predominated in both strains. A considerable amount (11 %) of PG at *m/z* 679.5 (29:0) was found in strain DSM 3257 (Fig. 5). Interestingly, among LPG, the most intense signal was identified for the ion at *m/z* 849 that was extracted from strain I′1a. The product-ion spectrum from MS2 on the [M–H]^−^ ion at *m/z* 849 observed in the negative-ion mode was dominated by an ion at *m/z* 145, which corresponded to deprotonated lysine. Fragmentation of the ion *m/z* 849 also yielded an intense fragment ion at *m/z* 241 and *m/z* 269, which corresponded to 15:0- and 17:0-carboxylate anions, respectively. These findings suggest that the compound that corresponded to the [M–H]^−^ ion at *m/z* 849 was 15:0/17:0 LPG (Fig. S2B).

### Fatty acid composition

A preliminary study of fatty acid methyl esters was carried out by GC/MS because the LC–MS/MS analysis had provided no data about the methyl branched fatty acyl chains or double bond positions and geometry (Mazzella et al. 2004).

The dominant fatty acids of the *B. subtilis* strains that we studied were 13-methyltetradecanoic (iC15:0) and 12-methyltetradecanoic (aC15:0) acids, followed by palmitic (C16:0), 14-methylhexadecanoic (aC17:0), 15-methyl-hexadecanoic (iC17:0) and myristic (C14:0) acids (Table 3). Branched chains, as well as iso- and anteiso- fatty acids, were the predominant components of lipids. Moreover, despite similar characteristics, strain I′1a could be distinguished from strain DSM 3257 by an increased percentage of aC15:0 and aC17:0, and a decreased amount of iC12:0 (Table 3).

**Table 3:** Fatty acid contents (%) of *B. subtilis* strains DSM 3257, DSM 3257 with added I′1a LPs extracts or I′1a after 48-h cultivation in LB-medium

Fatty acid	*B. subtilis* strain		
	DSM 3257	I′1a	DSM 3257 with added I′1a LPs extract
C13:0	10.53 ± 0.44	0.26 ± 0.06	4.63 ± 0.23
iC12:0	6.63 ± 0.15	1.21 ± 0.06	2.82 ± 0.09
C14:0	6.52 ± 0.16	2.28 ± 0.12	5.41 ± 0.47
iC15:0	31.88 ± 0.98	25.05 ± 1.16	26.37 ± 1.94
aC15:0	7.73 ± 0.56	33.94 ± 1.5	15.49 ± 1.02
C15:0	1.04 ± 0.06	–	1.05 ± 0.08
iC14:1	5.90 ± 0.18	3.42 ± 0.13	4.58 ± 0.35
C16:1	6.3 ± 0.11	1.39 ± 0.11	3.35 ± 0.18
C16:0	10.3 ± 0.44	11.18 ± 0.94	12.57 ± 0.98
C17:1	1.82 ± 0.14	1.13 ± 0.15	1.38 ± 0.09
iC17:0	7.81 ± 0.10	9.68 ± 0.19	16.28 ± 1.03
aC17:0	2.57 ± 0.24	10.24 ± 0.49	4.46 ± 0.32
C18:0	1.33 ± 0.28	0.41 ± 0.07	1.40 ± 0.11

Data represent mean ± SD

*a* anteiso branched fatty acid, *i* iso branched fatty acid

### Tolerance of the *B. subtilis* DSM 3257 strain to I′1a lipopeptides extracts

**Fig. 6:**
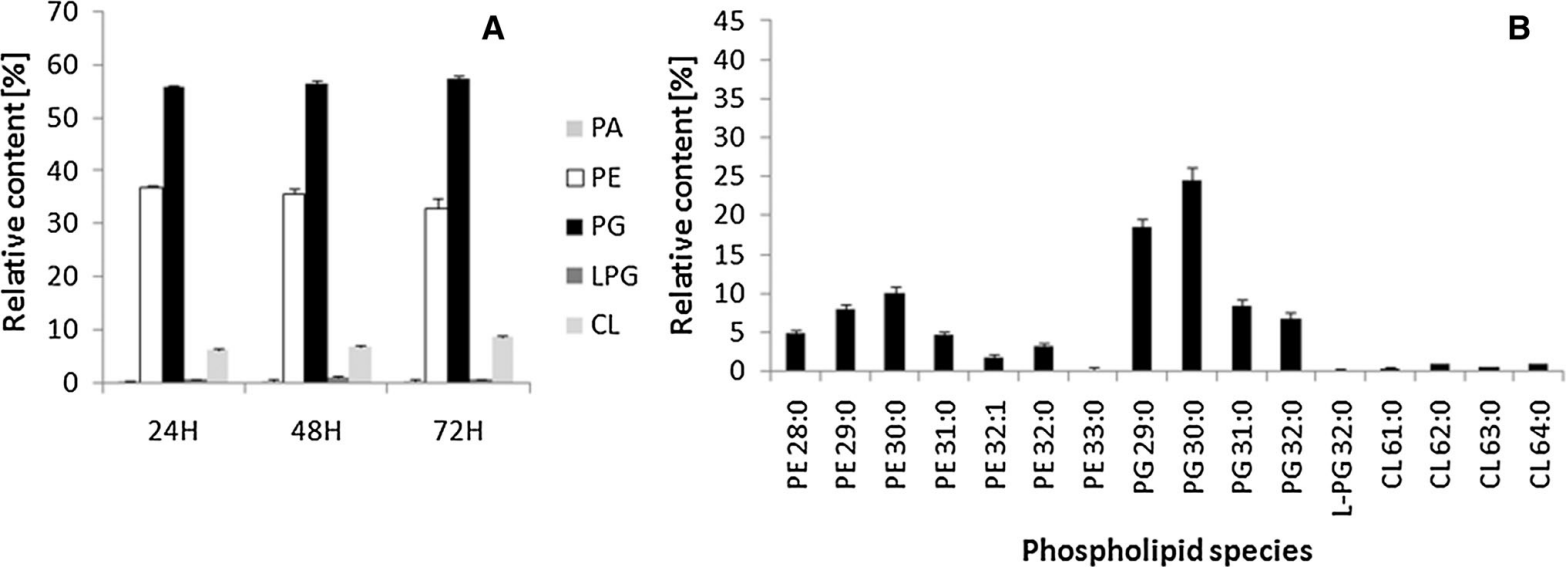
Relative abundance of phospholipids classes (**a)** and species (**b)** in *B. subtilis* DSM 3257 incubated with I′1a LPs extract. *PA* phosphatidic acid, *PE* phosphatidylethanolamine, *PG* phosphatidylglycerol, *LPG* lysyl-phosphatidylglycerol, *Cl* cardiolipin. Data represent mean ± SD (n = 3)

The growth kinetics of the studied culture with added LPs extract from *B. subtilis* I′1a was similar to the growth rate of the control culture (data not shown). However, the phospholipid profile of the bacteria strains differ from that of the phospholipids obtained from control strains (Fig. 6). Comparing samples from the DSM 3257 culture supplemented or not with I′1a extract, the strain exposed to a high concentration of lipopeptides had lower level of PE and an increased concentration of PG. After 3 days of culture, the PG-to-PE ratio increased from 1.54 to 1.77. Moreover, PGs at *m/z* 679.5 (29:0) and 693.5 (30:0) predominated in that strain. Interestingly, bacterial biomass exposed to I′1a extract was characterized by an increased percentage of aC15:0 and aC17:0 compared to samples without added extract (Table 3).

## Discussion

Lipopeptide biosurfactants from *Bacillus* species are well-known as one of the most efficient microbial surfactants (Singh et al. 2014). Cyclic LPs belonging to the surfactin, iturin, and fengycin families appear to be suitable candidates for bacterial eradication (Stein 2005). Currently, significant attention is being directed towards the antibacterial properties of surfactin. Mireles et al. (2001) observed that surfactin (at 100 µg) could reduce biofilm formation on urinary catheters by *Salmonella enterica*. A reduction in biofilm biomass formation in the presence of surfactin (at 66 µg/mL) was also observed for *Legionella pneumophila* (Loiseau et al. 2015).

The *B. subtilis* I′1a extract was the most active against the bacteria that we tested (both planktonic and sessile forms). This extract primarily contained iturin A and fengycin, which demonstrated a synergistic effect in the eradication of uropathogens. Rivardo et al. (2009) observed a huge impact of a mixture of lipopeptides from *B. subtilis* and *B. licheniformis* that belonged to the fengycin- and surfactin-like family of compounds on the inhibition of *E. coli* and *S. aureus* adhesion to an abiotic surface (they caused a reduction by 97 and 90 %, respectively). However, no apparent effect of these lipopeptides on the planktonic forms of tested bacteria was observed.

Cyclic LPs, especially surfactin and iturin, interact with the cytoplasmic membrane and disturb its integrity (Seydlová et al. 2013). However, data about the influence of lipopeptides on the lipid membrane content of biosurfactant producers is scarce.

Because of the amphiphilic nature of surfactin, iturin and fengycin points toward cellular membranes as the most probable site of their action (Meena and Kanwar 2015). However, the growth rate of the overproducer of LPs, I′1a, was found to be similar to that of strain DSM 3257. LP secretion did not impair the producer strain because the multiplication of bacteria continued in culture in parallel with biosurfactant accumulation.

Surfactin is one one of the most efficient surface active agents as it reduces the surface tension at the water–air interface from 72 to 27–30 mN/m (Sen 2010). By contrast, iturin reduces the surface tension of water to 43 mN/m (Jacques 2011). Surface tension measurements obtained in this study confirmed the presence of efficient surface active compounds that were produced by strains I′1a.

I′1a could synthesize high concentrations of iturin A. The amount of lipopeptide, expressed as a sum of iturin A homologues, reached 56 mg/L at 72 h of cultivation. Interestingly, these data were obtained without optimization of medium composition and conditions. Moreover, improved production of iturin A by *B. amyloliquefaciens* B128 up to 128 mg/L was described by Lin et al. (2007).

Using MS/MS methods, iturin A exhibited a high mass intensity at *m/z* 1043 and 1057 in their protonated forms and at *m/z* 1065 and 1079 as sodium adducts. An intensive signal of sodium adducts was also reported by others (Yang et al. 2015). In the time range of 3.0–3.5 min, peaks of fengycin variants were observed. The highest signals were identified at *m/z* 1457, 1471, 1485, 1513 and 1527. A similar mass intensity was described in other studies that used MALDI MS/MS (Pathak et al. 2014; Yang et al. 2015).

Phospholipids are major components of bacterial cell membranes. Therefore, while studying the possible mechanisms that allow the producer cells to survive exposure to high concentrations of LPs that can perturb membranes, this study investigated phospholipids. PG and PE are major components of the phospholipid profile for the studied *B. subtilis* species followed by smaller amounts of anionic CL and positively charged LPG. A similar profile of *B. subtilis* phospholipids has been reported by others (Griffiths and Setlow 2009; Sebastiani et al. 2012; Lobasso et al. 2013).

The lipid composition of DSM 3257 changed during growth, as CL rises at the expense of PG during culture. This phenomenon has also been described before by Lobasso et al. (2013) for *B. subtilis* cells, and it appears to be modulated by oxygen availability. However, for strain I′1a, conversion of PG in CL was not observed. It cannot be excluded that CL increases at the expense of PE. Tan et al. (2012) identified CL synthase (*clsC*). Unlike *clsA* and *clsB* (which use PG and CDP-diacylglycerol as substrates), *ClsC* used PE as a phosphatidyl donor to PG to yield CL.

When we compared the LP overproducer against strain DSM 3257, we observed that the significant reduction in *B. subtilis* phospholipid composition was associated with the PG-to-PE ratio. In bacteria, PE is cone shaped and favors the non-bilayer hexagonal phase, while in contrast PG, a cylindrical molecule, favors the bilayer (Dowhan et al. 2008). According to the computational study of Murzyn et al. (2005), it appears that the increase in the amount of PG in relation to PE allows for the stability and low permeability of the membrane to be maintained by increasing the average phospholipid headgroup area and presumably the chain order.

In all studied *B. subtilis* strains, the dominant phospholipid species included PG 30:0, which was also identified in *B. subtilis* by Gidden et al. (2009) using MALDI-TOF.

To characterize the role of possible modifications and structural adaptations of membrane lipids in lipopeptide producers, a fatty acids analysis was performed. In many *Bacillus* species branched-chain fatty acids are major cellular fatty acids (Kaneda 1991). A similar phenomenon was observed in the strains investigated in this present study. In bacteria, a change from the iso to anteiso configuration causes an increase in the fluidity of the membrane because of steric differences among these fatty acid species (Kaneda 1991). For C15 and C17 fatty acids, which are predominant in selected strains, transition temperatures of the single fatty acids were 51.7 and 60.2 °C for C15:0 iso and C17:0 iso, respectively. By contrast, temperatures reached 23.0 and 36.8 °C for C15:0 anteiso and C17:0 anteiso, respectively (Kaneda 1991). A visible increase in the anteiso series in strain I′1a, compared with DSM 3257, may indicate a marked increase in membrane fluidity (Unell et al. 2007).

An attempt was made to establish whether changes in membrane lipid and fatty acid profiles were linked to tolerance of the production of lipopeptide surfactants. Interestingly, the PG-to-PE ratio increased in DSM 3257 cultured with a lipopeptides extract of strain I′1a. Moreover, the anteiso series of fatty acids visibly increased in bacterial cultures and likely changed the membrane fluidity. After taking into account these results, it seems that the LP extract altered the lipid profile of DSM 3257 and resulted in changes that were similar to those described for cultures of strain I′1a.

## Conclusions

Our findings confirmed the high biological activity of iturin A and other lipopeptides synthesized by *B. subtilis* strain I′1a. Compared with the surfactin sample (extracted from DSM 3257 cultures) the mixture of LPs obtained from the I′1a culture exhibited a considerably higher inhibitory effect against both planktonic and sessile forms of uropathogens, which suggested potential applications of bacilli LPs in medical practice. Moreover, the profile of strain I′1a was characterized by an increased amount of anteiso fatty acids and a ten-fold higher ratio of PG-to-PE. Interestingly, lipopeptide extracts from strain I′1a added to the DSM 3257 culture increased the ratio of PG-to-PE and the amount of anteiso fatty acids in the bacterial strain.
